# Advances and challenges in predicting the impact of lymphatic filariasis elimination programmes by mathematical modelling

**DOI:** 10.1186/1475-2883-5-5

**Published:** 2006-03-28

**Authors:** Wilma A Stolk, Sake J de Vlas, J Dik F Habbema

**Affiliations:** 1 Department of Public Health, Erasmus MC, University Medical Center Rotterdam, P.O. Box 2040, 3000 CA Rotterdam, The Netherlands

## Abstract

Mathematical simulation models for transmission and control of lymphatic filariasis are useful tools for studying the prospects of lymphatic filariasis elimination. Two simulation models are currently being used. The first, EPIFIL, is a population-based, deterministic model that simulates average trends in infection intensity over time. The second, LYMFASIM, is an individual-based, stochastic model that simulates acquisition and loss of infection for each individual in the simulated population, taking account of individual characteristics. For settings like Pondicherry (India), where *Wuchereria bancrofti *infection is transmitted by *Culex quinquefasciatus*, the models give similar predictions of the coverage and number of treatment rounds required to bring microfilaraemia prevalence below a level of 0.5%. Nevertheless, published estimates of the duration of mass treatment required for elimination differed, due to the use of different indicators for elimination (EPIFIL: microfilaraemia prevalence < 0.5% after the last treatment; LYMFASIM: reduction of microfilaraemia prevalence to zero, within 40 years after the start of mass treatment). The two main challenges for future modelling work are: 1) quantification and validation of the models for other regions, for investigation of elimination prospects in situations with other vector-parasite combinations and endemicity levels than in Pondicherry; 2) application of the models to address a range of programmatic issues related to the monitoring and evaluation of ongoing control programmes. The models' usefulness could be enhanced by several extensions; inclusion of different diagnostic tests and natural history of disease in the models is of particular relevance.

## Review

### Introduction

Lymphatic filariasis is a mosquito-borne parasitic disease and an important cause of chronic morbidity in tropical countries. In 1998, the Global Programme to Eliminate Lymphatic Filariasis (GPELF) was initiated, aiming at the worldwide elimination of this parasitic disease as a public health problem [1]. The main strategy in the global programme is to interrupt transmission by annual population treatment with antifilarial drugs (diethylcarbamazine or ivermectin plus albendazole). In addition, morbidity management should reduce the suffering of patients who have chronic manifestations. Thirty-nine countries had started elimination programmes in 2004 [2] and this number is still growing.

The goal of elimination is ambitious. Past mass treatment programmes had varying degrees of success. In some areas transmission was apparently interrupted [3]. In other areas elimination was not achieved, in spite of long-term control programmes [4,5]. How strategic choices, and operational or biological factors contribute to success or failure is poorly understood. It is unknown which coverage and duration of mass treatment programmes (and possible additional measures) are required to achieve elimination and how this depends on the vector and parasite strain, endemicity level, and the drugs that are used. Mathematical models can help to clarify these issues and application of such models is considered important for support of GPELF [6].

Mathematical models help us to understand the complex transmission dynamics of parasitic diseases and are useful tools for planning and evaluation of control programmes [7,8]. So-called 'full transmission models', which relate the acquisition of new infections to the intensity and distribution of infection in a human population, can be used to predict the impact of interventions on transmission. Three such models have been developed for lymphatic filariasis. The first was developed for the evaluation of a specific vector control programme [9]. Two more recent models, called EPIFIL [10,11] and LYMFASIM [12], are more general and are both used to predict the long-term impact of mass treatment and assess elimination prospects.

This review describes recent progress in the modelling of lymphatic filariasis, focussing on EPIFIL and LYMFASIM. After a brief introduction of the processes involved in transmission and control of lymphatic filariasis, we compare the basic structure of these models and the parameter quantification. Relevant model predictions are compared and differences are discussed. We identify remaining challenges and future research priorities.

### Processes in lymphatic filariasis transmission and control

**Figure 1:**
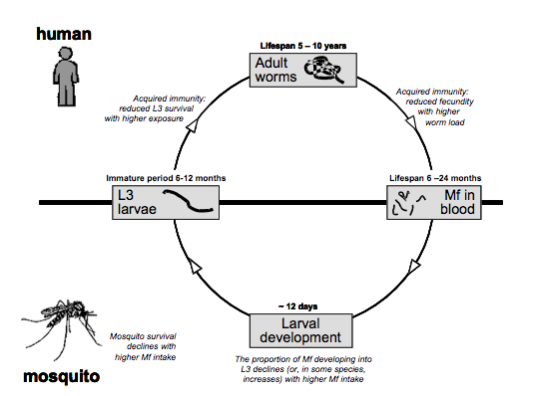
Transmission cycle of lymphatic filariasis with density-dependent mechanisms. This figure shows the life cycle of *Wuchereria bancrofti*, the main parasitic cause of lymphatic filariasis. The adult worms (macrofilariae) are located in the lymphatic system of the human host, where they live for 5–10 years [24, 37]. After mating with male worms, female worms can produce millions of microfilariae (mf), which can be found in the bloodstream and have a lifespan of 6–24 months [32]. A mosquito that takes a blood meal may engorge some mf. Inside the mosquito, mf develop in about 12 days into L3 stage larvae (L3), which are infectious to humans. When the mosquito takes another blood meal, the L3 can enter the human body and some will migrate to the lymphatic system and will develop into mature adult worms. The immature period lasts about 6–12 months [38]. Mf cannot develop into adult worms without passing through the developmental stages in the mosquito. Larval development and mosquito survival are density-dependent [17, 18]. Two possible mechanisms of acquired immunity are shown [20].

Models for lymphatic filariasis control basically describe the main biological processes involved in transmission (Figure 1). For prediction of the effects of intervention on transmission, it is of particular importance to take account of density dependence in these processes and to consider heterogeneities [13-15].

Density dependence is a biological term, which indicates that the growth rate of a population depends in a non-linear way on its density. We are most familiar with negative density-dependent mechanisms that limit the population growth (i.e. limitation, e.g. reduced survival probabilities due to crowding). Several of such limiting mechanisms are known to occur in lymphatic filariasis, particularly in the parasite development in mosquitoes. For example, there is a maximum number of microfilariae (mf) that can be engorged by mosquitoes and the number of L3 larvae that can survive. Consequently, the proportion of mf that develop into infectious L3 larvae in *Culex quinquefasciatus *does not increase linearly with mf intake, but saturates at higher levels [16,17]. In addition, the survival probability of mosquitoes may be reduced with their infection load [18]. This imposes a natural limit on infection intensity in mosquitoes. Density dependence may also occur in the opposite direction, facilitating transmission or population growth at higher infection intensities (i.e. facilitation). For example, the probability that a female worm mates with a male worm increases with higher worm burdens. Further, in some anopheline mosquito species, the probability that mf develop succesfully into L3 larvae increases when more mf are engorged [16], although at higher densities limiting mechanisms may eventually get the upper hand again. It is unknown to what extent density dependence, either limitation or facilitation, occurs in parasite development in humans (establishment, fertility and survival). Acquired immunity could limit one or more of these processes [19], but evidence for the operation of such immunity in lymphatic filariasis is inconclusive [20,21].

Density-dependent mechanisms as described above are important determinants of the elimination prospects. Due to such mechanisms, a reduction of one of the transmission determinants (e.g. mosquito biting rate, mf density in the blood) by control measures does not have a proportional effect on transmission rates and parasite abundance. Let us consider the example of limitation in the number of L3 developing in mosquitoes. When mf density in the human blood is brought down by mass treatment, mosquitoes will engorge less mf, but the probability that these mf develop into L3 increases. Therefore, the reduction in transmission is less than proportional to the reduction in mf density. The reverse is true in case of facilitation, so that the effect on transmission rates is more than proportional.

The term heterogeneity points at variation between individuals. Individuals differ for example in genetic background, nutritional status and behaviour, which may cause differences in exposure to mosquitoes, susceptibility to infection, and the survival, maturation and fecundity of parasites. Therefore, individuals may be predisposed to heavy or light infection, leading to an aggregated or overdispersed distribution of parasites (with a few hosts harbouring the majority of the parasites). This aggregation enhances transmission, because it increases the probability that female and male worms mate. Individuals also differ in compliance and responsiveness to treatment, which is important for the effect of mass treatment [22,23]. Heterogeneity may also occur in the parasite population, e.g. with respect to the life span and resistance to treatment.

### EPIFIL vs. LYMFASIM: model structure and quantification

The two available models for lymphatic filariasis transmission and control, EPIFIL and LYMFASIM, mainly differ in the amount of detail included. Specific variants of both models have been developed for *Wuchereria bancrofti *transmitted by *Culex quinquefasciatus*, using data from an integrated vector management control programme that was carried out in Pondicherry, India, from 1981 to 1985 [11,24]. These 'Pondicherry model variants' have been used for prediction. They are described below.

#### EPIFIL

EPIFIL simulates the average course of infection over age and time in a human population. The age-structure of the population is fixed, but its size is unspecified. Limitation in the transmission of infection by culicine mosquitoes is taken into account: the number of infectious L3 larvae that can develop in mosquitoes saturates at higher mf intensities. Acquired immunity is included as a second limiting mechanism: it is triggered by incoming L3 larvae and reduces the probability that new larvae develop into adult worms. The model takes account of heterogeneity that is introduced by age-related variation in exposure to mosquitoes: i.e. the exposure increases with age, until a maximum level is reached at the age of 9 years. A predetermined relationship between mf prevalence and intensity is used to translate predicted mf intensity levels into mf prevalences. The model can be used to simulate the impact of vector control, assuming that control measures reduce the mosquito biting rate. The effects of mass treatment can be simulated, assuming that a proportion of the population is treated with a drug with pre-specified efficacy; drugs may kill part of the present mf and adult worm and may reduce the mf production rate per adult worm.

The design of this population-based model is based on a general differential equation framework describing the dynamics of macroparasitic infections [13,19,25]. The model is deterministic, meaning that simulation output is always the same with fixed input specifications.

#### LYMFASIM

LYMFASIM simulates the acquisition and loss of worms over age and time in a discrete number of human individuals, using stochastic microsimulation. Individuals interact through biting mosquitoes and together they form a dynamic population, of which the size and age-structure may change over time. Like EPIFIL, LYMFASIM takes account of limitation in the proportion of engorged mf that develops into L3 larvae inside the mosquito and of acquired immunity in human hosts. Two model variants were developed for Pondicherry, which differed with respect to the type of acquired immunity: 'anti-L3' immunity is triggered by incoming L3 larvae and reduces the probability of successful adult worm establishment; 'anti-fecundity' immunity is triggered by the presence of adult worms and reduces the rate of mf production by female worms. By considering individual worms in individual hosts, the model inherently takes account of the declining mating probability of female and male worms with lower average infection intensities. Several sources of heterogeneity are considered in this model. This includes age-variation in exposure: exposure increases until a maximum is reached at about 20 years of age. Other factors contributing to heterogeneity are between-person variation in exposure (not age-related), and variation in inclination to participate in treatment programmes, the response to treatment, and the ability to develop immune responses. Parasites may vary with respect to their life span (about 10 years on average). Individual mf intensities are translated into the number of mf that would be counted in a 20 μl blood smear, taking account of random variability in these counts and reduced sensitivity of diagnostic tests at lower mf densities. The mf prevalence and mean mf intensity can be directly calculated from the smear counts, using data from all simulated individuals or specific subgroups. Similar to EPIFIL, LYMFASIM can simulate the impact of vector control by assuming that it reduces the mosquito biting rate. The model can simulate mass or selective treatment. In the first case, treatment is given to part of the of individuals, irrespective of their infection status; in the latter case, treatment is given only to a proportion of mf positives. Treatment of an individual may kill a proportion of mf and worms and may temporarily or permanently reduce the fertility of female worms. Treatment effects may vary between individuals.

This individual-based model uses the technique of stochastic microsimulation [26], which was earlier applied in the modelling of onchocerciasis transmission and control [27]. The stochastic nature of the model implies that there is variation in simulation outcomes, even if input specifications are exactly the same.

#### Parameter values

Table 1 gives the quantification of key biological model parameters. Both models were quantified for Pondicherry, but several assumptions were different. For example, EPIFIL quantified parameters for the adult worm lifespan, age-variation in exposure and the mf production per worm using information from literature and local data. LYMFASIM estimated these parameters by fitting the model to observed epidemiological data and arrived at somewhat different values. The quantification of the monthly biting rate was based on local data in both models, but it was much higher in EPIFIL than in LYMFASIM (5760 vs. 2200). EPIFIL's quantification was based on weekly mosquito catches that had been carried out in several sites in Pondicherry during the first few hours of the night. LYMFASIM's quantification was based on data from one single site where all night mosquito catches had been carried out. To compensate for the higher biting rate in EPIFIL, a lower value needed to be estimated for the proportion of inoculated L3 larvae that develops successfully into adult worms.

**Table 1:** Quantification of several key biological parameters in the EPIFIL and LYMFASIM model variants for Pondicherry, where *Wuchereria bancrofti *is transmitted by *Culex quinquefasciatus*. Values in bold-face were estimated indirectly by fitting the model outcomes to observed epidemiological data; other values were quantified directly from literature, theory, and analysis of local data

Parameter	EPIFIL	LYMFASIM	
		
		Anti-L3 immunity	Anti-fecundity immunity
*Parasite lifecycle*			
Average adult worm life span in years	8 ^a^	**10.2 **^b^	**11.8 **^b^
Average mf life span in months	10 ^a^	10 ^c^	10 ^c^
Premature period in months	-	8	8
*Exposure variation by age*			
Exposure at age zero as fraction of maximum exposure	0	**0.26**	**0.40**
Age in years at which maximum exposure is achieved	9	**19.1**	**21.3**
*Density dependence in mosquitoes*			
Maximum number of L3 larvae that can develop in mosquitoes at high mf intensities	6 ^d^	6.6 ^e^	6.6 ^e^
*Acquired immunity*			
Duration of acquired immunity in years	lifelong	**9.6 **^f^	**11.2 **^f^
*Other parameters*			
Monthly biting rate	5760	2200	2200
Proportion of L3 larvae in mosquitoes that enters the human host when a mosquito bites	0.414*0.32 = 0.13	0.1	0.1
Proportion of inoculated L3 larvae that develops successfully into adult worms (x10^-3^)	**0.113**	**1.03 **^g^	**0.42**
Mf production per worm	2	**0.61 **^h^	**4.03 **^h, i^

- Not considered in the model; mf, microfilaria.

^a ^Assuming an exponential distribution.

^b ^Assuming a Weibull distribution with shape parameter α = 2.

^c ^Assuming an exponential distribution, approximated with discrete time steps.

^d ^Exponential saturating function with initial increase from zero = 0.047.

^e ^Hyperbolic saturating function with initial increase from zero = 0.09.

^f ^Period in which the strength of the immune response is halved in the absence of boosting.

^g ^In the absence of anti-L3 immunity.

^h ^In the presence of at least 1 male worm, scaled to the number of mf per 20 μl peripheral blood.

^i ^In the absence of anti-fecundity immunity.

**Figure 2:**
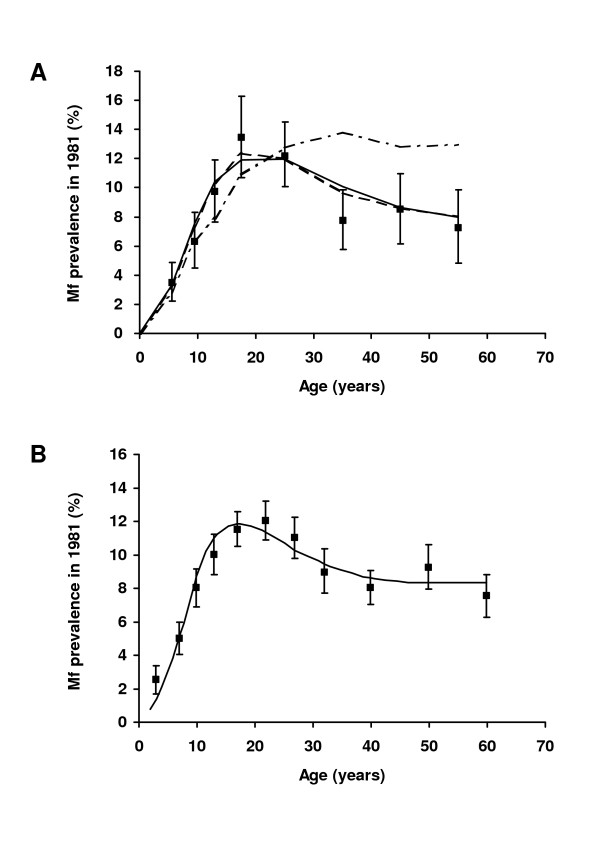
Comparison of model predictions of microfilaraemia prevalence by age with observed data, before the start of vector control (1981) in Pondicherry, India. (A) LYMFASIM predictions for models with anti-L3 immunity (solid line), anti-fecundity immunity (dashed line), and a model variant without immunity (dot-dashed line); the latter model did not fit the data and was therefore rejected. Source: [24]. (B) EPIFIL predictions of a model with acquired immunity. Source: [11]. Symbols in both graphs indicate the observed prevalence levels with corresponding confidence intervals.

Both models accurately mimicked epidemiological data from Pondicherry even though different assumptions were made. Figure 2 illustrates the good fit of both models to the precontrol (1981) data from Pondicherry.

### EPIFIL vs. LYMFASIM: predictions

Both EPIFIL and LYMFASIM have been used to predict the impact of control measures and assess prospects for elimination by mass treatment [28-31]. We focus here on model predictions of the coverage and duration of annual mass treatment programmes required for elimination. All published predictions were based on the Pondicherry variants of the models, although acquired immunity was left out of the model in the EPIFIL predictions. LYMFASIM's predictions were based on the models with anti-L3 or anti-fecundity immunity, with a population size of about 3000–4500 individuals.

**Figure 3:**
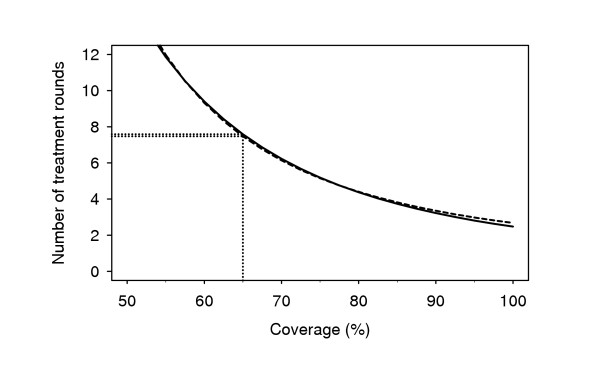
LYMFASIM predictions of the coverage and number of yearly mass treatment rounds with ivermectin that are required for lymphatic filariasis elimination in Pondicherry, India. Precontrol microfilaraemia prevalence was assumed to be 8.5%. Elimination was said to occur if zero microfilaraemia prevalence is reached 40 years after the start of treatment, with 99% probability. A single treatment with ivermectin (200 μg/kg) was assumed to sterilize 77% of female worms permanently and to kill all microfilariae. Results are shown for two variants of the LYMFASIM model for Pondicherry, differing with respect to the assumed immune mechanism (solid line – anti-L3 immunity; broken line – anti-fecundity immunity). Source: [30].

**Figure 4:**
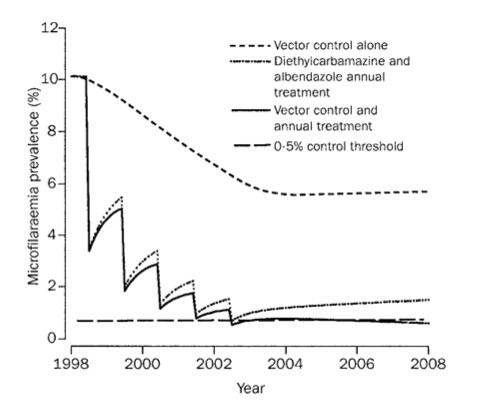
EPIFIL predictions of the impact of mass treatment, vector control and their combination on trends in microfilaraemia prevalence. Predictions were made with the EPIFIL simulation model as quantified for Pondicherry (but ignoring acquired immunity), assuming a precontrol microfilaraemia prevalence of 10%. The plot shows the impact of mass treatment alone (5 rounds of annual mass treatment with diethylcarbamazine + albendazole, with a coverage of 80%), vector control alone (assuming a 90% reduction in biting rate during 5 years), and the combination of the two. Reprinted from [28], with permission from Elsevier.

From the predictions of both models we can conclude that it is possible to eliminate lymphatic filariasis by yearly mass treatment, but that the number of treatment rounds largely depends on coverage, precontrol mf prevalence and the macrofilaricidal effects of drugs. This is illustrated in Tables 2 and 3, and Figure 3. In many situations, the predicted number of yearly treatment rounds required for elimination was higher than 4 to 6, which was hoped to be sufficient when GPELF was initiated. As an alternative to longer programmes, one might consider more frequent mass treatment (e.g. half-yearly) or the additional application of vector control (Figure 4).

**Table 2:** LYMFASIM predictions of the number of annual mass drug treatment rounds required to achieve elimination in an area like Pondicherry, with 99% probability. Results are shown for four different drugs or drug combinations and two coverage levels. Predictions are based on the anti-L3 variant of the model for Pondicherry, with a precontrol microfilaraemia prevalence of 8.5%. Elimination is defined as zero microfilaraemia prevalence 40 years after the start of treatment. Source: [31]

	Assumed treatment effects (proportion killed)		Predicted number of rounds for elimination, with coverage	
Drug(s)	adult worms	microfilariae	65%	80%

Ivermectin + albendazole	35%	100%	10	6
Diethylcarbamazine	50%	70%	8	5
Diethylcarbamazine + albendazole	65%	70%	6	4
Doxycycline	80%	0%	6	4

**Table 3:** EPIFIL and LYMFASIM predictions of the number of yearly mass treatment rounds that is required to reach a 0.5% microfilaraemia prevalence threshold. Results are shown for mass treatment with a combination diethylcarbamazine plus albendazole, and for various endemicity and coverage levels. The combination treatment is assumed to kill 55% of all adult worms and 95% of the microfilariae, and to interrupt the microfilaria production for 6 months. EPIFIL's predictions were made with a model without acquired immunity. LYMFASIM predictions, from the model with anti-L3 immunity, were added for comparison for an average pretreatment microfilaraemia prevalence of 10%. Details of the variability between LYMFASIM runs are included in the lower part of the table. The EPIFIL predictions were reprinted from [28], with permission from Elsevier

	Coverage			
Pretreatment mf prevalence	60%	70%	80%	90%

EPIFIL				
2.5%	7	6	5	4
5%	9	7	6	5
10%	10	8	7	6
15%	12	9	8	7
LYMFASIM ^a^				
10% (p5 – p95: 8.8% – 11.4%)	10	8	6	5
Details of the 100 simulation runs on which the LYMFASIM estimations were based:				
Average mf prevalence, 1 year after last treatment round (p5 – p95)	0.49 (0.29–0.73)	0.39 (0.25–0.58)	0.42 (0.24–0.64)	0.33 (0.22–0.47)
Number of runs (%) with zero mf prevalence 40 years after start of treatment, out of the total number that				
- had achieved the 0.5% threshold	35/51 (69%)	79/86 (92%)	62/70 (89%)	90/97 (93%)
- had NOT achieved the 0.5% threshold	16/49 (33%)	8/14 (57%)	18/30 (60%)	1/3 (33%)

^a ^Based on the average trend in microfilaraemia prevalence of 100 simulation runs.

The predictions for Pondicherry-like situations indicate that elimination can be achieved within a reasonable timeframe. In fact, the required time period is shorter than the mean adult worm lifespan. This is possible, because the antifilarial drugs are thought to have strong macrofilaricidal or sterilizing effect [23,32-34]. Moreover, not all worms need to be killed or sterilized in order to achieve elimination. The prevalence just needs to be brought under a threshold or breakpoint level, below which the acquisition rate of new worms is lower than the death rate of existing worms (by treatment or natural death), so that the worm population will eventually die out. Clearly, the quantitative predictions should be interpreted with some care. Achieving elimination would for example be more difficult, if the macrofilaricidal effects of treatment are lower, if the adult worms live longer, if there is stronger aggregation of worm-burdens, or if density-dependent mechanisms operate that enhance parasite transmission at low infection intensities. In spite of these uncertainties, the predictions give important information on the determinants of elimination.

The predictions of EPIFIL and LYMFASIM cannot be compared directly, because the original publications reported results for different treatment regimens, with different assumptions on efficacy of the drugs, and different precontrol mf prevalence levels. Further, different criteria for elimination were used: in EPIFIL elimination was assumed to occur if the mf prevalence after treatment was below 0.5%; in LYMFASIM elimination was defined as a zero mf prevalence 40 years after the start of control in 99% of the runs. To allow better comparison of the models, we did a series of additional simulations with LYMFASIM for mass treatment with the combination of diethylcarbamazine plus albendazole, using the same assumptions on drug-efficacy and the same criterion for elimination as in published EPIFIL predictions (Table 3).

The two models come to comparable conclusions regarding the number of treatment rounds required to bring mf prevalence below 0.5%, although LYMFASIM's predictions are slightly more optimistic than EPIFIL's at higher coverage levels. This finding of nearly equal predictions is not straightforward. The LYMFASIM model contains several assumptions and mechanisms, which, relative to EPIFIL, limit the impact of the intervention on transmission: 1) a longer adult worm life span (10 vs. 8 years); 2) acquired immunity; 3) heterogeneities in exposure to mosquitoes, in compliance to mass treatment, and in adult worm life span. However, the limiting effect of these assumptions and mechanisms on the impact of mass treatment is apparently counteracted by the enhancing effect of a reduced mating probability of worms at lower average worm burdens in LYMFASIM.

### Criteria for elimination

The choice of elimination criterion influences predictions of the efforts required for elimination. EPIFIL's predictions were based on the assumption that transmission will not continue when the mf prevalence falls below 0.5%. The choice for this threshold is somewhat arbitrary in the absence of evidence from the field. LYMFASIM's predictions were based on the eventual reduction of mf prevalence to zero (40 years after the start of control) in 99% of the simulation runs and depend strongly on the model's accurate reflection of transmission dynamics at low infection levels. It is interesting to investigate the relationship between the outcomes of the two models and the employed elimination criteria. See Table 3.

Let's consider the situation with a precontrol mf prevalence of 10% and 70% coverage per treatment round. Both LYMFASIM and EPIFIL predicted that 8 rounds of mass treatment were sufficient to bring the average mf prevalence below 0.5%. For LYMFASIM, this conclusion was based on the average trend in 100 runs, but there was variation between the runs. The precontrol mf prevalence was 10% on average, but varied between runs (5^th ^– 95^th ^percentile (p5 – p95): 8.8% – 11.4%). The post-treatment mf prevalence (1 year after the 8^th ^treatment) was 0.39% on average, with p5 – p95 = 0.25% – 0.59%. The 0.5% threshold was reached in 86/100 runs, but elimination (zero mf prevalence 40 years after the start of control) was only found in 79 of these 86 runs. The 0.5% threshold was not reached in 14/100 runs, but elimination occurred nevertheless in 8. Overall, elimination was achieved in 87% of the runs in this example. One or two extra rounds of mass treatment would be required to be 99% certain of elimination.

The numbers are much less favourable in the simulations with 60% coverage, so that the extra number of treatment rounds required to be 99% certain of elimination would be larger. More extensive simulation studies are required to study how the threshold mf prevalence level, below which elimination almost always occur, depends on local transmission dynamics and mosquito biting rates, immigration of parasite carriers or invasion of infected mosquitoes, heterogeneities and population size.

### Challenges in application of models for support of GPELF

The published model predictions are helpful in planning and design of elimination programmes. However, major challenges remain if modelling is to be used more widely for decision support in GPELF. Firstly, the models need to be quantified and validated for regions with different transmission dynamics. Secondly, a wide range of programmatic issues remains to be addressed.

#### Quantification and validation of models for other regions

The first major challenge lies in the quantification and validation of model variants for different regions. The discussed models were both quantified for transmission of *W. bancrofti *by *Culex quinquefasciatus *and tested against data from Pondicherry [11,24]. Predictions for Pondicherry cannot simply be generalized to other areas, because transmission dynamics and therefore also elimination prospects are different due to different vector-parasite combinations and endemicity levels. Region-specific model variants are required.

The development of new model variants requires region-specific data. Although the value of some parameters can be assumed to be independent of the area of interest, several others must be requantified. Because density dependence in the relationship between mf density in the human blood and the number of L3 larvae developing in mosquitoes strongly determines elimination prospects, accurate quantification of this relationship is particularly important. Unfortunately, though, few data are available for the different mosquito species that can transmit the infection [35]. Especially for the anopheline mosquito species, which are responsible for transmission in large parts of Africa, more field research is needed. Data on mosquito biting rates and heterogeneity in exposure are similarly important and difficult to find.

It is crucial to test the validity of new model variants against epidemiological data. The models reflect current knowledge of the biology of infection, but some uncertainty remains in the model structure (e.g. is there density dependence in parasite development in the human host or not?) and the quantification of specific model parameters (e.g. parasite life span). It is therefore crucial to continue testing the validity of existing and new model variants against epidemiological data. Different types of data help to test different parts of the model. For example, age-specific data on infection prevalence and intensity may help to determine the role of acquired immunity and age-related processes [36]. Epidemiological trends during vector control are especially informative on the adult worm life span [24,37]. Trends during mass treatment may give information on the effects of drugs on worm survival and productivity. And trends after cessation of control may help to determine whether density-dependent mechanisms have appropriately been included in the model. Such tests may lead to adaptation of the model structure or parameter quantifications.

Some work has already been done to prepare models for use in other areas. The LYMFASIM model has been applied to age-patterns observed in an area in South-East India that has the same vector-parasite combination and presumably the same transmission dynamics as Pondicherry. This led to the development of new model variants with less strong or no immunity (Subramanian, unpublished data). EPIFIL was also tested against data from this area to test whether the model could match observed trends during mass treatment with current assumptions regarding efficacy of drugs and local transmission dynamics [28]. LYMFASIM is currently being adapted for use in Africa (Stolk, unpublished data).

#### Programmatic issues to be addressed

The second major challenge for lymphatic filariasis models lies in their application: several programmatic issues remain to be addressed.

So far, the models have been used to estimate the number of treatment rounds required for elimination and to examine the benefit of additional measures in specific situations. This work needs to be extended to determine more extensively where and when additional measures are required, and which measures are most cost-effective. Outcomes will be helpful to develop more specific guidelines for elimination programmes.

Besides these strategic issues, several urgent monitoring and evaluation issues need to be addressed. Any elimination programme faces the following questions: is the programme on track towards elimination or is programme adjustment required? Can we safely stop mass treatment without a risk of recrudescence? To better address these issues, the lymphatic filariasis transmission models should be extended with newer diagnostic tools. Most important is inclusion of the card test for antigen detection, which is widely used in the monitoring and surveillance of ongoing control programmes. This will help in the development of more precise criteria for when to stop mass treatment and how to monitor after cessation of control. It will also allow better calibration of the model to local situations, by taking better account of earlier achieved reduction in mf prevalence and intensity. This, in turn, will improve the accuracy of predictions for a specific situation. Other extensions of the model may also have to be considered, such as immigration of parasite carriers or invasion of infected mosquitoes or the development of resistance to available drugs.

Although discussion until now focused on the elimination of transmission, this goal may be too ambitious for some areas. The focus may shift to reducing the public health problem without explicitly aiming at eliminating infection. To address this with the models, more attention is required for the development of disease. Simple mechanisms of disease development are included in both models, but natural history of disease has received little attention in published work until now.

## Conclusion

Important advances have been made in the modelling of lymphatic filariasis transmission and control. However, with the rapidly expanding Global Programme to Eliminate Lymphatic Filariasis there is increasing demand for model-based support of decision-making. Huge challenges remain: models need to be quantified and validated for specific regions and a wide range of programmatic issues remain to be addressed. The models' usefulness could be enhanced by several extensions; inclusion of different diagnostic tests and natural history of disease in the models is of particular relevance. A close link between modellers and elimination programmes is crucial for successful prediction of the impact of lymphatic filariasis elimination programmes: the models can be improved based on new data collected for evaluation of the elimination programmes, while the programmes can benefit from more precise guidelines and predictions that are specific for the local situation concerned.

## Abbreviations

mf = microfilariae

GPELF = Global Programme to Eliminate Lymphatic Filariasis

## Competing interests

The author(s) declare that they have no competing interests.

## Authors' contributions

WS drafted the manuscript. SV and DH helped to draft the manuscirpt. All authors read and approved the final manuscript.
